# First Description of the Early Stage Biology of the Genus *Mygona*: The Natural History of the Satyrine Butterfly, *Mygona irmina* in Eastern Ecuador

**DOI:** 10.1673/031.011.0105

**Published:** 2011-01-17

**Authors:** Harold F. Greeney, Lee A. Dyer, Tomasz W. Pyrcz

**Affiliations:** ^1^ Yanayacu Biological Station and Center for Creative Studies, Cosanga, Ecuador, c/o 721 Foch y Amazonas, Quito, Ecuador; ^2^ Biology 0314, University of Nevada, Reno, 1664 N. Virginia St., Reno, Nevada 89557; ^3^ Zoological Museum of the Jagiellonian University, Ingardena 6, 30–060 Kraków, Poland

**Keywords:** Andes, bamboo, *Chusquea*, cloud forest, larva, Poaceae, pupa

## Abstract

The immature stages and natural history of *Mygona irmina* Doubleday (Lepidoptera: Nymphalidae: Satyrinae: Pronophilina) from northeastern Ecuadorian cloud forests are described based on 17 rearings. The dwarf bamboo, *Chusquea c.f. scandens* Kunth (Poaceae, Bambusoidea) is the larval food plant. Eggs are laid singly on the bottom side of mature host plant leaves. Larvae take 102–109 days to mature from egg to adult. Adults are encountered most frequently on sunny days, flying rapidly over areas dominated by their food plant or feeding on the ground at mammal feces. Males are often encountered inside large forest gaps near patches of bamboo guarding perches in the mid-canopy.

## Introduction

The generic limits of the subtribe Pronophilina (Nymphalidae, Satyrinae) are still unclear and recent authors (e.g. Viloria 1998, 2003; Lamas et al. 2004; Pyrcz 2004; Peña et al. 2006) have used several different arrangements since Miller's (1968) treatment of the Satyrinae. The genus *Mygona* (Thieme) is one of the genera that have been included consistently in the Pronophilina, and is considered most closely related to *Lasiophila* (C. and R. Felder) based on adult morphology (Pyrcz 1999). While D'Abrera (1988) recognized seven species of *Mygona*, subsequent revisions consider the genus to include only three species (Lamas et al. 2004; Pyrcz 2004). However, all these occur allopatrically, are morphologically similar, and could be considered to represent one widespread, polytypic species. *Mygona irmina* Doubleday (Lepidoptera: Nymphalidae: Satyrinae: Pronophilina) differs from all other congeners in the large, pale blue dorsal hindwing median patch, a pattern that is rather unusual among within the subtribe Pronophilini. The only somewhat similarly patterned sympatric species is *Oxeoschistus leucospilos* Staudinger. In the past, another species was associated with the genus *Mygona*, originally described as *Pronophila propylea* Hewitson (Adams, 1986) and currently placed in the genus *Proboscis* Thieme (Pyrcz, 2004). *Proboscis propylea* replaces *M. irmina* parapatrically at higher elevations, a fact noticed by Adams (1985). *Mygona irmina* (Figure 8c) flies at elevations of *ca.* 1800–2500 m and is distributed southward from Venezuela, occurring in the Cordillera de la Costa (its type locality) in the Cordillera de Merida and the Sierra de Perija, throughout Colombia, and in Ecuador. In Ecuador it is found on the western slopes of the Andes in the north and on the eastern slopes in the north and central Andes. The southern distributional limit is Rio Pastaza valley. South of this distribution it is replaced by its ally *M. poeania* (Hewitson). To date there are no recognised subspecies of *M. irmina*, yet some geographic races are fairly distinct (TW Pyrcz unpublished observations). Nominotypical individuals are characterised by the noticeably smaller size compared to other populations, while those from the Chocó slopes of the Colombian Western Cordillera are generally larger and bear the largest blue patch on the dorsal hind wing. Specimens reared and collected as adults at the Yanayacu Biological Station (YBS) do not differ markedly from other Ecuadorian populations.

Almost nothing is known of the early stages of any genus of Pronophilina with only a few scattered records of food plant associations (e.g. Adams and Bernard 1981; Adams 1986; Miller 1986; DeVries 1987; Pyrcz and Wojtusiak 1999) and larval descriptions (Schultze 1929), mostly within the genus *Pedaliodes* (Butler, 1867) (Pelz 1997; Heredia and Viloria 2004; Greeney et al. 2009). There is no published information on the life cycle of any species of *Mygona*, and here a description of the early stages of *Mygona irmina* is presented based on rearings in northeastern Ecuador.

## Materials and Methods

All rearing and field investigations were carried out at the Yanayacu Biological Station and Center for Creative Studies (00° 35.949 S, 77° 53.403 W), located in Napo Province, in the Andes of northeastern Ecuador. The study site is located approximately 5 km west of the town of the town of Cosanga, adjacent to Cabañas San Isidro, and encompasses about 2000 hectares of primary cloud forest bordered by cattle pasture and other disturbed habitats. A single oviposition event was observed and this egg was reared through to eclosion. Subsequently, three mid-second instars were reared through to eclosion of the imago. Landslides are common in the steep terrain surrounding YBS, most of which are dominated by *Chusquea* bamboo. Large patches of bamboo also form in flatter areas, especially along streams. For more complete site descriptions see Greeney (2008) and Valencia (1995). Larvae were collected at elevations ranging from 2000–2200 m, and they were reared in glass jars at the ambient research lab, located at 2150 m. In the laboratory, fresh food plant leaves were added as needed, and frass and old host plant leaves were removed daily. Larval measurements were made the day prior to molting.

## Results

### Larval behavior

The dwarf bamboo, *Chusquea c.f. scandens* Kunth (Poaceae, Bambusoidea) is the larval food plant of *M. irmina* in the studied area. Larvae were found in patches of bamboo along roads, streams, and in land slides. First and second instars rest and feed near the tip of mature leaves. Feeding damage is such that one margin of the leaf apex is left intact, and larvae rest near the tip of this thinned portion of the leaf where they are very cryptic. Late second, third, and fourth instars rest on the dorsal surface of leaves, often traveling to adjacent leaves to feed. Their green coloration makes them extremely cryptic on mature leaves, as they rest with their head tipped forward and scoli appressed to the leaf surface. Fifth instar larvae were not observed in the field, but their distinctive, dark coloration suggests they rest somewhere other than the leaves or stems of the host plant.

They most likely rest in dark areas of rotting vegetation trapped in tangles of the host plant or on rotting portions of the stem near the ground or perhaps off the plant.

### Egg (Figures 1A–B)

**Figure 1.**
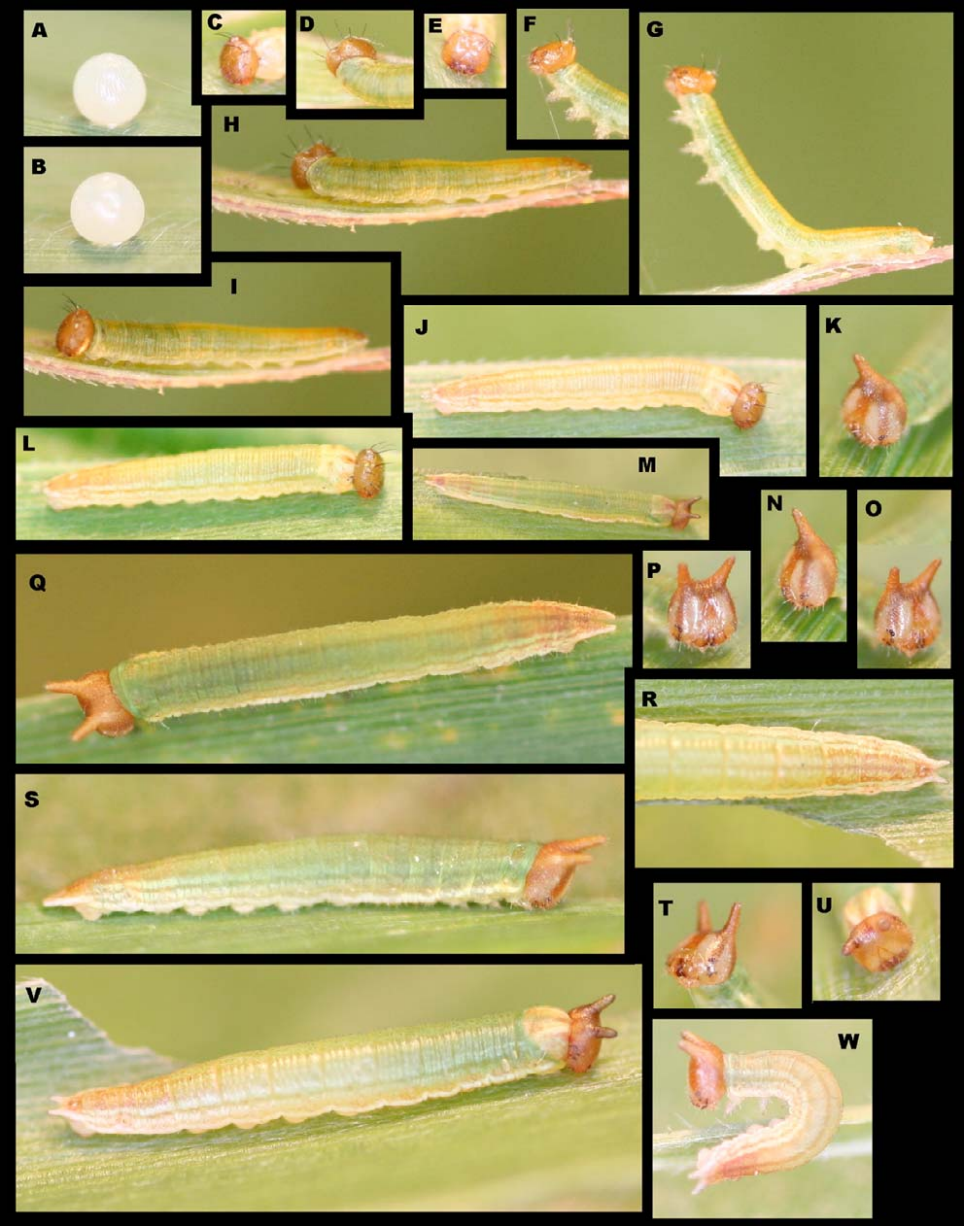
Immature stages of *Mygona irmina* at Yanayacu Biological Station, Napo Province, 2100 m, Ecuador: (A-B) freshly laid egg; (C-J and I) first instar; (K and M-W) second instar. High quality figures are available online.

**N = 1; approx. 1 mm diameter; 13 days.** Egg round, white, appear smooth but minute, irregular, vertically oriented striations visible under a dissecting scope (Figure 1A). Eggs are laid singly (n =1). Upon emergence, larvae consume the entire egg shell.

### First instar (Figures 1C–J, 1L)

**N** = **1; 2–5.5 mm; 20 days.** From a cephalad view, the head capsule nearly square and narrowing slightly dorsally and with a shallow epicranial crease, light brown and shining, bearing sparse, forward-curved, dark setae laterally and dorsally, longest dorsally; body white upon emergence with very pale red-brown, thin longitudinal stripes on dorsum and sides, body widest around Al, tapering gradually posteriorly to very small, widely separated caudal tails, roughly round in cross-section, body bare except for sparse, short, dark setae, 1–3 per segment ventrolaterally, longest on T1, and A1O; dorsal prothoracic shield thin, weakly scoleritized. Later in first stadia larvae appearing greenish from ingested leaf material and with longitudinal stripes more prominent. The terminal 4–5 abdominal segments bear a rust-brown wash dorsally. Just prior to molting, the dorsum appears highlighted with whitish stripes (Fig. 1J), and caudal tails become slightly more noticeable, but remain short.

### Second instar (Figures 1K, 1M–W)

**N** = **4; to 10.5 mm; 13 days.** Head capsule roughly square, cream colored with distinct brown vertical stripes anterior-laterally extending upward to completely brown epicranium and scoli. Posteriorly head paler brown, entire head finely pitted and reticulated with sparse, short, pale setae basally; epicranium bears two widely-spaced, prominent, rounded, forward-angled horns. Body round in cross-section and similar in coloration to late first instar, but with rust-brown wash more pronounced on terminal abdominal segments, A10 now bears pronounced, outward-angled, conical tails forming a distinct V when viewed from above (Fig. 1R).

### Third instar (Figure 2)

**N = 4; to 15.5 mm; 10–11 days.** Head similar in coloration to second instar but posterior surface of the head scoli now paler, head now distinctly granulated appearing bumpy; body now bears a thin white ventro-lateral stripe running the length of body, extending onto caudal tails, most visible from T1-A3, A3 also bears a pair of small, white, subdorsal spots; body more flattened, widest around A2 and tapering to two tightly appressed caudal tails. Later in instar A3 spots become more pronounced and separate into three smaller spots. The ventrolateral white stripe becomes more distinct and a pair of small white spots becomes visible subdorsally on A2. The T1 and A8 spiracles are dark and clearly visible.

### Fourth instar (Figures 3–4)

**N = 10; to 24.5 mm; 11–13 days.** Head similar to that described for third instar, head horns curving slightly outward and distinctly pale posteriorly; body similar in form to third instar with lateral expansion prominent on A2–4, caudal tails longer, held tightly appressed and distinctly pointed, body coloration similar to third instar but with ventrolateral white stripe very pronounced and with A1–A3 bearing elongated black patches subventrally; subdorsal markings on A3 no distinctly compose of a tight line of three white spots, A2 and A4 now bearing single white spots in the same position as those on A3, rusty wash on terminal abdominal segments still present early in stadium but confined to the basal two-thirds of caudal tails later, apical third of caudal tails dark brown to black late in stadium, late in stadiumT1-A2 develop an indistinct, subdorsal, pale line, fading posteriorly.

**Figure 2.**
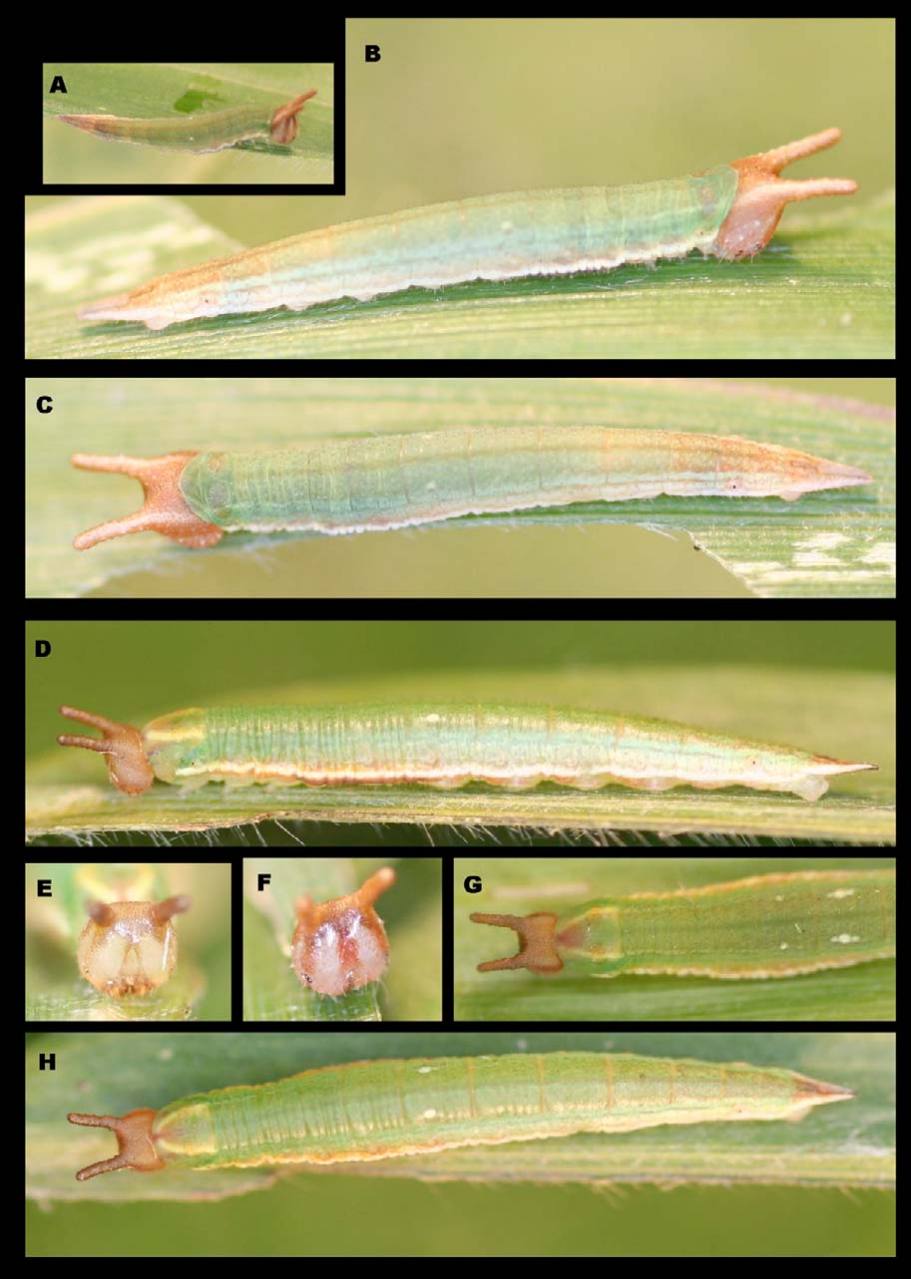
Immature stages of *Mygona irmina* at Yanayacu Biological Station, Napo Province, 2100 m, Ecuador: (A-H) third instar. High quality figures are available online.

**Figure 3.**
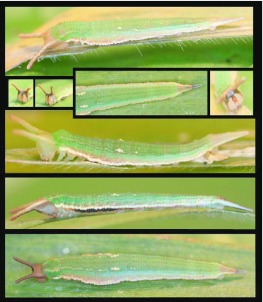
Immature stages of *Mygona irmina* at Yanayacu Biological Station, Napo Province, 2100 m, Ecuador: (A-H) fourth instar. High quality figures are available online.

### Fifth instar (Figures 5–6)

**N = 13; to 32 mm; 16**–**17 days including pre-pupa.** Fifth instars are very distinct. Head now dark brown to black, darkening (Figures 5A, B) slowly from pale green (Figures 5A 6G) to black (Figures 5C, 6B–D, 6F) after molting, scoli well developed, curving forward, heavily reticulated (Figures 6D, 6F–G), late in stadium head appears lighter brown (Figure 5d); body shape similar to fourth instar but widest portion now at A2 and with caudal tails proportionately shorter, thicker, more triangular, but still held tightly appressed (Figure 6C), larval coloration changes significantly during stadium (Figure 5). Immediately after molting to fifth instar, larvae are various shades of brown, green, and pale blue dorsally and dorsolaterally and with shades of white and pink spiracularly to ventrolaterally (Figure 5A). Recently molted larvae have the white spots subdorsally on A2–A4 very prominent but fading with age. Over the course of 12–24 h larvae become darker overall, with blue areas becoming more distinct, especially on the supraspiracles on A1–3. Green areas darken, brown areas become black, and pink areas become purplish (Figure 5B). After ca. 24 h larvae have darkened to mostly black with no sign of any white markings except for prominent, thin white line ventrolaterally from T1-A4 or A5 (Figures 5C, 6A). Dorsum of mid-stadium larvae marked with light brown forming a pair of thin, broken lines subdorsally to A4 or A5 and a mid-dorsal line from T3–A7 or A8. Segments A2–A7 distinguished by indistinct, forward-opening chevrons dorsally. Late in Stadium, near pupation, body ground color becomes predominantly brown with dark brown areas forming distinct dorsal chevrons highlighted with small green flecking (Figure 5D).

**Figure 4.**
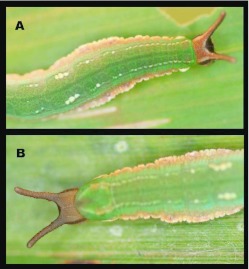
Immature stages of *Mygona irmina* at Yanayacu Biological Station, Napo Province, 2100 m, Ecuador: (A-B) fourth instar. High quality figures are available online.

**Figure 5.**
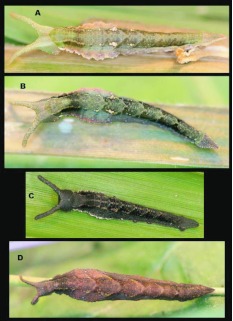
Immature stages of *Mygona irmina* at Yanayacu Biological Station, Napo Province, 2100 m, Ecuador: (A) recently molted fifth instar; (B) fifth instar ca. 8 h after molting; (C) fifth instar ca. 24 h after molting; (D) mature fifth instar. High quality figures are available online.

### Pre-pupa (Figures 7A–B)

**N = 13; ca. 24 mm; 3 days.** Pre-pupal larvae appear similar to late fifth instars, but become more uniformly brown to black, with most markings fading except for subdorsal white line.

### Pupa (Figures 7C–F, 8A–B, 8D)

**N = 12; ca. 18 mm; 19–22 days.** Pupa hangs downward and is similar to the nymphal genus *Adelpha.* Ground color light brown with dense, minute darker markings and a few sparse light green flecks, mostly on abdomen posterior to wing pads (Figures 7D–E); shape was robust with well- developed, flattened, leaf-like projections, a pair dorsolaterally on anterior abdominal segments (Figure 7E) and a single projection mid-dorsally on anterior portion of thorax; head bears a pair of short, flattened, rounded, downward-curved projections (Figures 7F, 8D); cremaster same color as body, stout, attached to a white silk pad.

**Figure 6.**
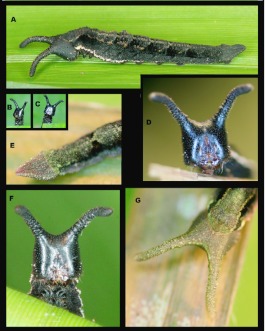
Immature stages of *Mygona irmina* at Yanayacu Biological Station, Napo Province, 2100 m, Ecuador: (A-G) fifth instar. High quality figures are available online.

### Adult behavior (Figure 8C)

Adult *Mygona irmina* are rarely observed in the area. They prefer to fly only on sunny days, generally from 1000–1400 h. Both sexes were most frequently encountered while feeding at excrement or urine at forest edges, or in sunny gaps within the forest. When not feeding they fly rapidly along areas of *Chusquea* bamboo with a rapid, erratic flight pattern, and were easily confused with the sympatric *Oxeoschistus* leucospilos (Satyrinae, Pronophilina). They can be separated on the wing, however, by slightly more erratic flight and by the bluish sheen to the dorsal, hindwing, discal patch. Overall, the flight of *Mygona* differs from other similarly-sized pronophilines in being slower and broken with occasional gliding. From mid-late afternoon on sunny days males frequently seen guarding perches in the sub-canopy over areas of disturbance. They use a series of regular perches from which to fly out and chase any passing butterflies of similar size. Interestingly, this sort of aggressive territorial defense has not been observed in other localities (TW Pyrcz, unpublished observations).

**Figure 7.**
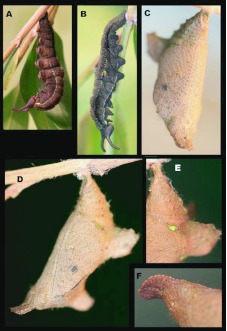
Immature stages of *Mygona irmina* at Yanayacu Biological Station, Napo Province, 2100 m, Ecuador: (A-B) pre-pupal larva; (C-F) pupa. High quality figures are available online.

Only a single oviposition event was observed. At 1145 h a female *M. irmina* paused briefly on the upper surface of a *Chusquea c.f. scandens* Kunth leaf overhanging a forested stream. She curled her abdomen under and deposited a single egg on the lower surface of a mature leaf. The entire oviposition lasted only several seconds, after which she flew rapidly from the area.

## Discussion

Like many Pronophilina with known host-associations (e.g. Schultze 1929; DeVries 1987; Pyrcz and Wojtusiak 1999; Pyrcz 2000; Heredia and Viloria 2004; Greeney et al. 2009) *M. irmina* feeds on *Chusquea* bamboo, a widespread and dominant genus of bamboo in the Andes (Judziewicz et al. 1999).

**Figure 8.**
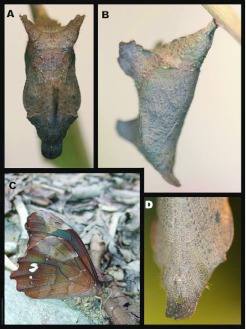
Pupa and imago of *Mygona irmina* at Yanayacu Biological Station, Napo Province, 2100 m, Ecuador: (A-B, D) pupa; (C) adult feeding at urine roadside. High quality figures are available online.

However, the dearth of published life history information for other members of the subtribe Pronophilina provides few examples with which to compare the morphology of *M. irmina* early stages. The species-rich and widespread genus *Pedaliodes* is the best-documented group with respect to early stages (Schultze 1929; Pelz 1997; Heredia and Viloria 2004; Greeney et al. 2009). While Schultze (1929) illustrates various aspects of the larval stages of several other pronophiline genera, there are no modern figures with which to compare. Based on our own experience rearing many genera of neotropical Nymphalidae (including many Satyrinae), the final instar and pupa of *M. irmina* appears fairly unique within the Satyrinae. Most notably, *Mygona* appears to be the only genus whose larvae bear curved head scoli. *Mygona* shares long head scoli with *Corades, Junea*, and *Lymanopoda* and has head scoli that are widely separated as in *Lymanopoda, Corades, Pedaliodes*, and *Daedalma* (Schultze 1929; Greeney et al. 2010; Pyrcz et al. 2011). These latter two genera, however, have greatly reduced head scoli in relation to other described pronophiline larvae (Heredia and Viloria 2004; Greeney et al. 2009; Pyrcz et al. 2011). Similarly, *Mygona* differs from other genera in the form of its caudal tails. Like *Corades, Junea*, and *Lymanopoda, Mygona* has caudal tails which are held tightly appressed rather than separated as in *Pedaliodes* and *Daedalma* (Schultze 1929; Heredia and Viloria 2004; Greeney et al. 2010; Pyrcz et al. 2011). The caudal tails of *Mygona* are intermediate in length between the longer tails of *Corades, Junea*, and *Lymanopoda*, and the shorter, more conical tails of *Pedaliodes* and *Daedalma.*

Life history traits are well known to be phylogenetically informative in many taxa (e.g. Eickwort and Sakagami 1979; Zyskowski and Prum 1999; Funk et al. 2004), and larval lepidopteran morphology and behavior are frequently used for developing and testing phylogenetic hypotheses (e.g. Devries et al. 1985; Brown and Freitas 1994; Penz 1999; Freitas et al. 2002). With continued uncertainty about the inter-generic relationships within the Pronophilina (e.g. Pyrcz 2004; Peña et al. 2006), life histories for further members of this group are needed. As pointed out by Freitas and Brown (2004), larval characteristics of the Nymphalidae will likely prove invaluable, in combination with other characters, in the ongoing effort to sort out the evolutionary history of this group. It is hoped that this contribution stimulates others to publish their observations on the life histories of this and other taxa of increasingly threatened Neotropical organisms.
